# Functional Transforming Growth Factor-β Receptor Type II Expression by CD4^+^ T Cells in Peyer's Patches Is Essential for Oral Tolerance Induction

**DOI:** 10.1371/journal.pone.0027501

**Published:** 2011-11-07

**Authors:** Rebekah S. Gilbert, Ryoki Kobayashi, Shinichi Sekine, Kohtaro Fujihashi

**Affiliations:** 1 The Immunobiology Vaccine Center, The Department of Microbiology, The University of Alabama at Birmingham, Birmingham, Alabama, United States of America; 2 The Immunobiology Vaccine Center, The Department of Pediatric Dentistry, The University of Alabama at Birmingham, Birmingham, Alabama, United States of America; 3 Department of Preventive Dentistry, Osaka University, Suita, Osaka, Japan; Albany Medical College, United States of America

## Abstract

Our previous studies have shown that Peyer's patches (PPs) play a key role in the induction of oral tolerance. Therefore, we hypothesized that PPs are an important site for Transforming Growth Factor (TGF)- β signaling and sought to prove that this tissue is of importance in oral tolerance induction. We found that expression of TGF-β type II receptor (TGFβRII) by CD4**^+^** T cells increases and persists in the PPs of normal C57BL/6 mice after either high- or low-dose feeding of OVA when compared to mesenteric lymph nodes (MLNs) and spleen. Approximately one-third of these TGFβRII^+^ CD4**^+^** T cells express the transcription factor Foxp3. Interestingly, the number of TGFβRII^+^ CD4**^+^** T cells in PPs decreased when OVA-fed mice were orally challenged with OVA plus native cholera toxin (CT). In contrast, numbers of TGFβRII^+^ CD4**^+^** T cells were increased in the intestinal lamina propria (iLP) of these challenged mice. Further, these PP CD4^+^ TGFβRII^+^ T cells upregulated Foxp3 within 2 hours after OVA plus CT challenge. Mice fed PBS and challenged with OVA plus CT did not reveal any changes in TGFβRII expression by CD4**^+^** T cells. In order to test the functional property of TGFβRII in the induction of oral tolerance, CD4dnTGFβRII transgenic mice, in which TGFβRII signaling is abrogated from all CD4**^+^** T cells, were employed. Importantly, these mice could not develop oral tolerance to OVA. Our studies show a critical, dose-independent, role for TGFβRII expression and function by CD4**^+^** T cells in the gut-associated lymphoid tissues, further underlining the vital role of PPs in oral tolerance.

## Introduction

Oral tolerance is a function of the mucosal immune system by which the host is protected from deleterious immune responses to innocuous gut antigens (Ags) [1], [2]. Large doses of Ag or prolonged exposure to small doses of Ag induce a state of mucosal and systemic unresponsiveness that is characterized by reduced Ag-specific IgG and helper T cell responses in the presence of protective S-IgA antibody (Ab) production [1]-[3]. When oral tolerance is disrupted, allergy and inflammatory bowel diseases can occur. Conversely, it has been proposed that harnessing oral tolerance can be an effective means of treating various diseases, from allergy to autoimmunity [1], [4]-[9].

Although dendritic cells have been shown to be involved both directly and indirectly in the induction of oral tolerance [10]-[16], it is primarily agreed that oral tolerance is established and maintained at the T cell level [5], [17], [18]. The magnitude of the dose of Ag determines how the tolerance is mediated. Large doses of Ag are understood to induce anergy–a failure to respond to the Ag–and/or deletion of Ag-specific T cells, while small recurrent doses of Ag lead to the development of Ag-specific T regulatory cells (Tregs) which in turn suppress surrounding T cells by the production of inhibitory cytokines, such as TGF-β1 and IL-10 [1], [2], [19]-[22]. In addition to these mechanisms, recent studies have suggested that anergy is also important in small-dose oral tolerance [13] and vice versa, that active suppression can play a role in large-dose oral tolerance [13], [23].

TGF-β1 plays important roles in the induction and maintenance of tolerance. In the absence of IL-6, TGF-β1 induces the expression of Foxp3 in naïve CD4^+^ T cells *in vitro* [19], [24]-[26] and *in vivo* [25], [27]. Further, TGF-β1 has been shown to be necessary for the maintenance of Foxp3 expression in adaptive CD4^+^ CD25^+^ Tregs [28]. In addition to its roles in adaptive Treg differentiation and function, TGF-β1 suppresses Ag-specific effector T cells *in vitro* and *in vivo* [19], [25], [29].

TGF-β1 is recognized by a type I-type II hetero-oligomeric receptor [30]. TGF-β receptor type II (TGFβRII) binds TGF-β1 and activates the type I TGF-β receptor through the kinase region of its cytoplasmic tail, initiating the TGF-β1 response [30]. Point mutations in the kinase domain of TGFβRII abrogate the TGF-β1 signal [31]. Further, deletion of the kinase domain of the receptor through the use of a dominant-negative form of TGFβRII has been used to study the effects of TGF-β1 signal abrogation in many cell types, including mammary cells [32], osteoblasts [33], skin cells [34], and T cells [35]. In the latter, mice that express the dominant-negative TGFβRII protein on the surface of their T cells exhibit a phenotype very similar to that of TGF-β1 knockout mice in that they develop a lethal lymphoproliferative autoimmune syndrome [35]. Although these mice have thymus-derived natural Tregs, their effector T cells ultimately escape suppression [36].

Peyer's patches (PPs) play key roles in oral tolerance. Our previous studies showed that the presence of PPs was required for oral tolerance to proteins to occur [37]. Thus, PP-null mice fed a large dose of OVA and subsequently challenged systemically developed OVA-specific Abs and helper T cell responses [37]. In addition to this, targeted delivery of Ag to PPs using M-cell targeting protein s1 fused with OVA greatly reduces the amount of Ag needed to induce tolerance by up to 1,000 fold [38]. Others have shown that Ag-specific CD4^+^ CD25^+^ T cells with suppressive functions can be cloned from PPs of orally tolerized mice [23]. Despite these findings, the topic of a PP requirement in oral tolerance induction remains controversial. Subsequent studies of oral tolerance induction in PP-null mice showed that oral tolerance could be achieved [39], and that only the removal of the MLN prior to tolerization could prevent oral tolerance induction [40]. Early studies in germ-free mice linked the impairment of oral tolerance induction in these animals to the reduced number T cells in their PPs [41]. Yet, more recent studies found that adoptively transferred Ag-specific T cells isolated from high- and low-dose Ag-fed germ-free recipients proliferated the same as those from specific pathogen-free animals [42], suggesting again that PPs play a secondary role to MLNs.

The present study aims to provide further evidence for the vital role of PPs in early events in the induction of oral tolerance. We provide evidence that PPs are uniquely populated with T helper cells that are equipped to respond to TGF-β1 and may therefore contribute to the suppressive environment necessary for tolerance initiation. This study is the first to examine the expression of TGF-β1 receptors in mucosal and systemic inductive tissues in the early time points during the induction of oral tolerance.

## Materials and Methods

### Ethics Statement

All experiments involving mice were performed in accordance with both NIH and the University of Alabama at Birmingham (UAB) Institutional Animal Care and Use Committee (IACUC) guidelines. UAB IACUC gave specific approval for all procedures involving mice; Animal protocol number 100908212.

### Mice

Six- to 8-wk-old female C57BL/6 mice were purchased from the Frederick Cancer Research Facility (National Cancer Institute, Frederick, MD). Five-wk-old CD4dnTGFβRII mice and wild-type littermates were obtained from an in-house colony maintained at the Immunobiology Vaccine Center, the University of Alabama at Birmingham (UAB). Original transgenic breeder pairs were obtained from Dr. Richard Flavell at the Yale University School of Medicine. Upon arrival, mice were housed in microisolators, maintained in horizontal laminar flow cabinets, and provided sterile food and water as part of a specific-pathogen free facility at UAB. All of the mice used in these experiments were free of bacterial and viral pathogens.

### Induction of oral tolerance and oral challenge procedure

To establish oral tolerance to a high dose of oral protein, mice were gastrically intubated with 30 mg of OVA (Sigma-Aldrich) dissolved in 0.25 ml of PBS; control mice received PBS only. To establish low-dose oral tolerance, mice were continuously fed 1 mg/ml of OVA *ad libitum* in drinking water for 7 days; control groups received water only. To determine TGFβRII expression after oral challenge, mice were challenged intragastrically (i.g.) 7 days after tolerization with 1 mg of OVA plus 10 µg of native cholera toxin (CT; List Biological Laboratories, Inc., Campbell, CA) as mucosal adjuvant in 0.25 ml sterile PBS (OVA plus CT). To examine the induction of oral tolerance to OVA, transgenic and wild-type mice were challenged i.g. with OVA plus CT on days 7, 14 and 21 after an initial 30 mg dose of OVA or PBS as control [43].

### Sample collection and cell isolation

Plasma and fecal samples were collected one week after the final challenge with OVA plus CT. Fecal extracts were isolated from homogenates of feces in sterile PBS (1 g/ml). Mononuclear cells were isolated from spleen and mesenteric lymph nodes (MLNs) by a mechanical dissociation method using gentle teasing through stainless steel screens as described previously [38], [43]. Peyer's patches (PPs) were carefully excised from the small intestinal wall and dissociated using the neutral protease enzyme collagenase type IV (0.5 mg/ml; Sigma-Aldrich) in RPMI 1640 (Cellgro Mediatech, Washington, DC) containing 2% fetal bovine serum (FBS; Atlanta Biologicals, Lawrenceville, Georgia) to obtain single-cell preparations [38], [44]. Mononuclear cells in the iLP were isolated after removal of PP and intraepithelial lymphocytes from the small intestine using a combination of enzymatic dissociation and discontinuous Percoll gradients (Pharmacia Fine Chemicals, Uppsala, Sweden). Mononuclear cells in the interface between the 40% and 75% layers were removed, washed and resuspended in complete medium (RPMI 1640, Cellgro Mediatech, Washington, DC) containing 10% FCS, 1% L-glutamine, 10 mM HEPES, 100 U/ml penicillin, 100 µg/ml streptomycin, and 40 µg/ml gentamicin. [38], [44].

### OVA-specific Ab assays

OVA-specific Ab levels in plasma and fecal extracts were determined by ELISA as previously described [38], [43]-[45]. Endpoint titers were expressed as the last dilution yielding an optical density at 415 nm (OD_415_) of > 0.1 units above background control values. Mononuclear cells obtained from mucosal and systemic lymphoid tissues were subjected to an ELISPOT assay to detect numbers of OVA-specific Ab-forming cells (AFCs) per 10^6^ total lymphoid cells [38], [43], [45].

### DTH responses

Ag-specific DTH responses were measured 7 days after the last oral challenge with OVA plus CT as described above. Briefly, PBS (20 ml) containing 10 µg OVA was injected into the left ear pinna of the mice, whereas the right ear pinna received PBS as control. Ear swelling was measured 24 h later with a dial-thickness gauge (Ozaki Manufacturing, Tokyo, Japan). The DTH response was expressed as the increase of ear swelling after OVA injection minus the swelling in the control site [37].

### Flow cytometric analysis

Mononuclear cells from mucosal and systemic tissues were isolated at various time points and preincubated with purified CD16/32 mAb (Fc Block, 2 µg/ml; BD Pharmingen, San Diego, CA). Samples were then stained with a combination of FITC-labeled anti-human TGFβRII mAb (clone #25508; R&D Systems, Minneapolis, MN), allophycocyanin (APC)-conjugated anti-CCR9 or -CD4, PE-tagged anti-CD8a, -CD4, or -LPAM, and biotin-conjugated anti-CD3 mAbs (BD Pharmingen, San Diego, CA) followed by PerCP-Cy™5.5-conjugated streptavidin (BD Pharmingen, San Diego, CA). CD4dnTGFβRII mice over-express a non-functional TGF-βRII molecule on all CD4^+^ T cells; therefore CD4^+^ T cells were isolated from MLN and PP of CD4dnTGFβRII mice and stained as described above for use as positive controls for the staining of FITC-hTGFβRII mAb (Fig. S1). Intracellular cytokine staining was performed to determine the presence of IL-10 and TGF-β1. After 4 h stimulation with PMA-ionomycin, the cells were surface stained as above, fixed and permeablized using a Foxp3 staining buffer kit (eBioscience, San Diego, CA). Cells were then stained intracellularly with PE-conjugated anti-IL-10 (BD Pharmingen, San Diego, CA) or anti-hTGF-β1 (R&D Systems, Minneapolis, MN). In some experiments, intracellular Foxp3 expression was detected using a Foxp3 staining buffer kit and PE-conjugated anti-hFoxp3 (eBioscience, San Diego, CA).

### Statistical Analysis

The results are expressed as the mean ± one SEM. Non-parametric data were analyzed using an unpaired Mann-Whitney *U* test using InStat software for Macintosh by GraphPad. Parametric data were analyzed using two-way ANOVA using Prism software for Windows by GraphPad. *p* values < 0.05 were considered significant, and < 0.001 were considered highly significant.

## Results

### TGFβRII expression is induced and persistent in the Peyer's patches after OVA feeding

**Figure 1:**
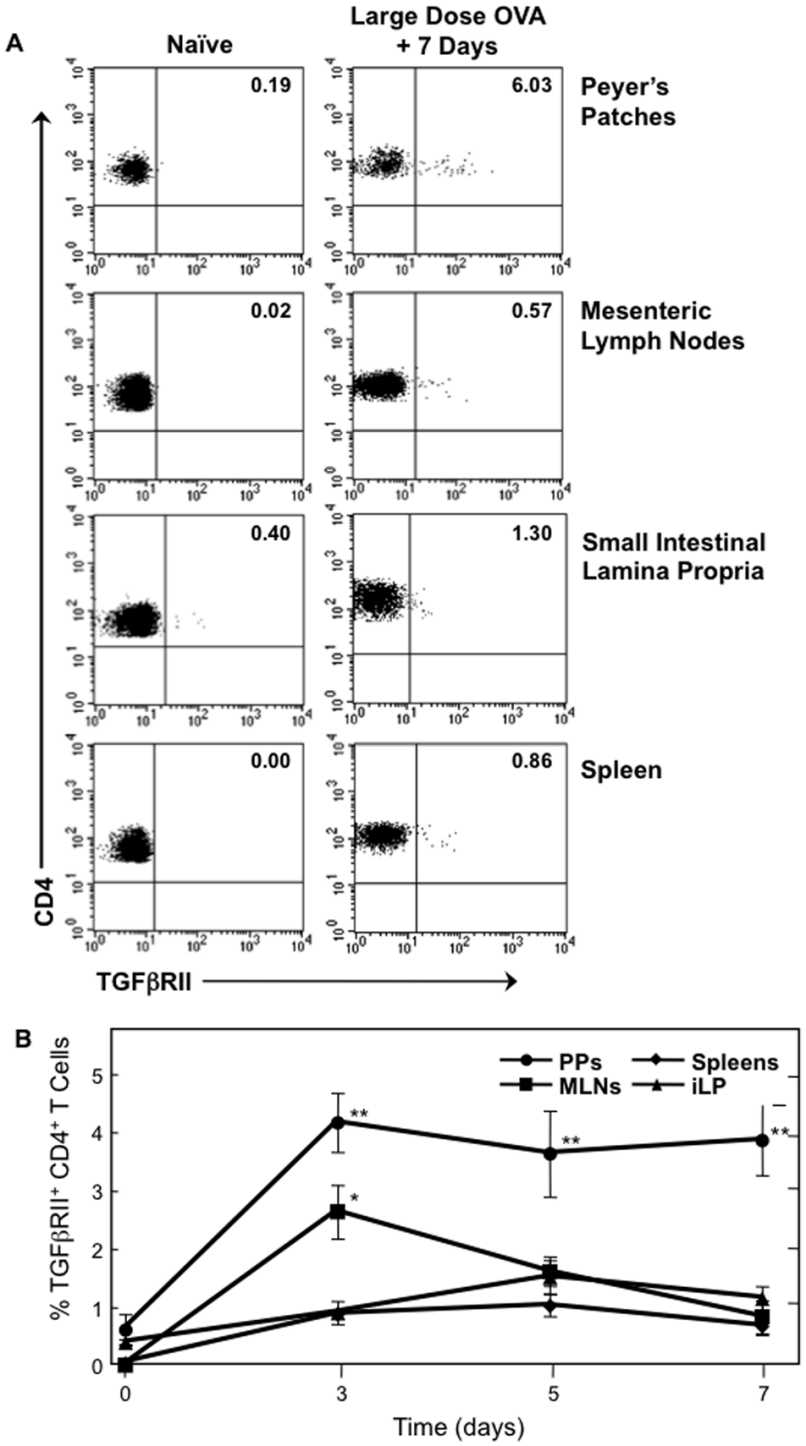
Effect of large-dose OVA feeding on the occurrence of CD4^+^ TGFβRII^+^ T cells. Mononuclear cells were isolated from PPs, MLNs, iLP and spleens of naïve C57BL/6 mice (0) and at 24 h time points after 30 mg of OVA feeding. Cells were then stained with FITC-conjugated anti-hTGFβRII, APC-labeled anti-CD4, and biotin-tagged anti-CD3 mAbs followed by PerCP-Cy™5.5-conjugated streptavidin. Samples were subjected to flow cytometry analysis by FACSCalibur®. *A.* Plots are representative of naïve tissues and tissues analyzed 7 days after OVA feeding. *B*, Time course of CD4^+^ TGFβRII^+^ T cells in PPs, MLNs, iLP and spleens 3, 5 and 7 days after 30 mg of OVA feeding, *n* = 20 mice per time point, *p<0.05 or **p<0.001 when compared to sham-tolerized control group.

We initially determined the expression of the TGFβRII on CD4^+^ T cells in the PPs, MLNs, small intestine lamina propria (iLP) and spleen of C57BL/6 mice using flow cytometry. We found that TGFβRII is not expressed on CD4^+^ T cells of naïve mice (data not shown). We next assessed the kinetics of this expression after feeding large or small doses of oral OVA or PBS as a negative control. Interestingly, TGFβRII expression by CD4^+^ T cells increases within 24 h after feeding a 30 mg dose of OVA when compared to those in tissues from PBS-fed mice. PPs of mice fed 30 mg OVA had significantly higher frequencies of CD4^+^ TGFβRII^+^ T cells than mice fed PBS alone, 4% versus 0.6%, respectively. These frequencies were maintained for seven days after Ag feeding, the point at which an oral challenge protocol would begin (Fig. 1A and 1B). In contrast, the frequencies of CD4^+^ TGFβRII^+^ T cells in MLNs, iLP, and spleen fluctuated and returned to basal levels over this time course, and did not exceed 2% of the CD4^+^ T cell population.

**Figure 2:**
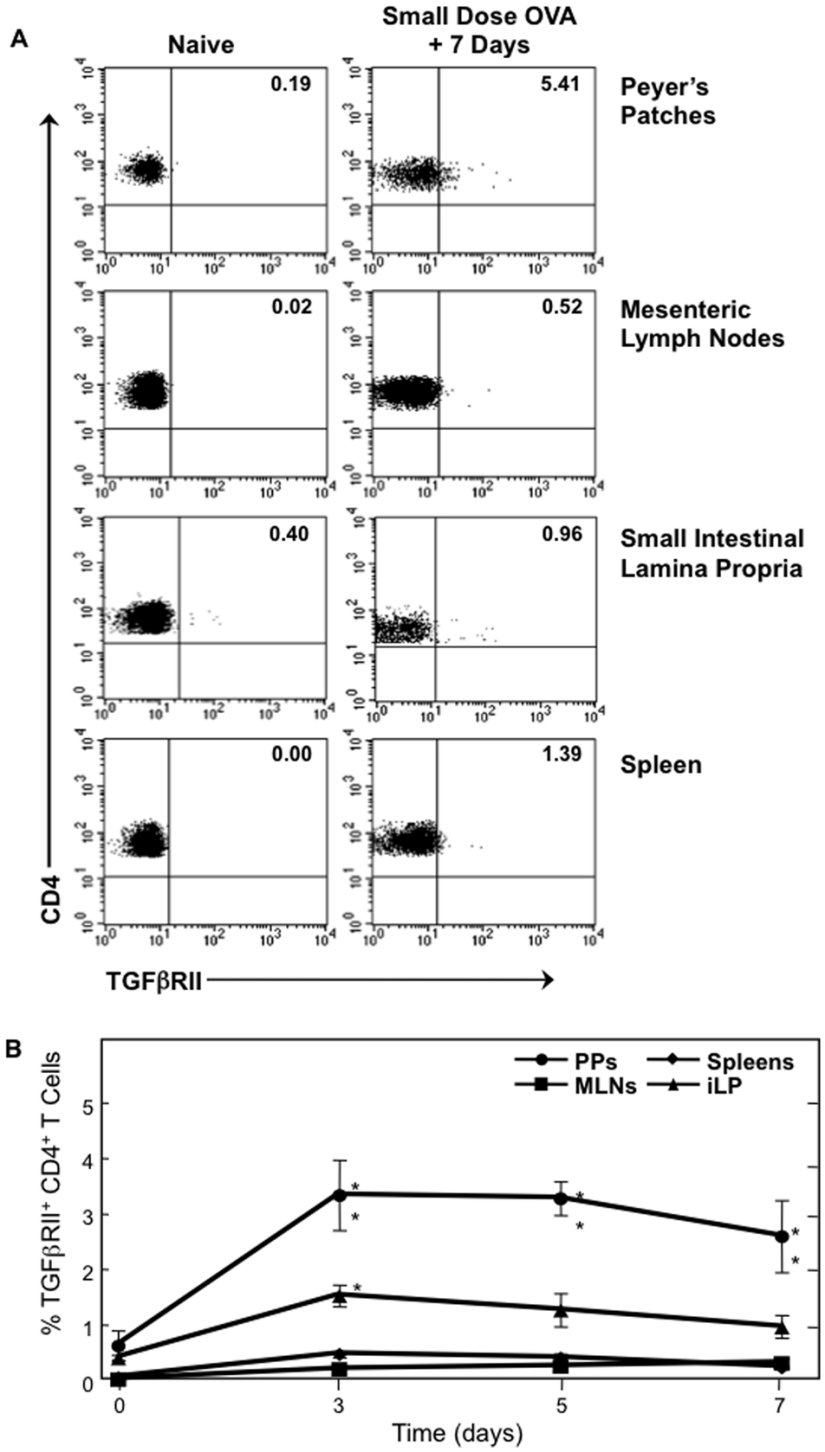
Effect of small-dose OVA feeding on the occurrence of CD4^+^ TGFβRII^+^ T cells. Mononuclear cells were isolated from PPs, MLNs, iLP, and spleens of naïve C57BL/6 mice (0) and at time points during *ad libitum* OVA feeding (1 mg/ml). Cells were then stained with FITC-conjugated anti-hTGFβRII, APC-labeled anti-CD4, and biotin-tagged anti-CD3 mAbs followed by PerCP-Cy™5.5-conjugated streptavidin. Samples were subjected to flow cytometry analysis by FACSCalibur®. *A.* Plots are representative of naïve tissues and tissues analyzed 7 days after beginning OVA feeding. *B*, Time course of CD4^+^ TGFβRII^+^ T cells frequency in PPs, MLNs, iLP and spleens 3, 5 and 7 days after 30 mg of OVA feeding, *n* = 10 mice per time point, **p<0.01 when compared to sham-tolerized control group.

When fed a low dose of OVA (1 mg/ml in drinking water for 7 days), a similar pattern of TGFβRII expression was observed, with the PP CD4^+^ TGFβRII^+^ T cell frequency reaching 3.3% of all CD4^+^ T cells (Fig. 2A and 2B). This frequency was significantly higher than in PPs of mice that drank only water. Further, the frequencies of CD4^+^ TGFβRII^+^ T cells in MLNs and spleens of small-dose recipients were not significantly different from those of water-fed control mice. The frequency of these cells in the iLP was significantly higher than water-fed mice at only one time point after beginning the small-dose regimen. These data indicate that TGF-β1 signaling is occurring and is sustained to a higher degree in PPs for initiating the potential development of Treg cells when compared to systemic and other mucosal lymphoid tissues during large- and small-dose oral tolerance induction.

### Expression of TGFβRII on CD4^+^ T cells decreases after oral challenge with OVA plus CT

**Figure 3:**
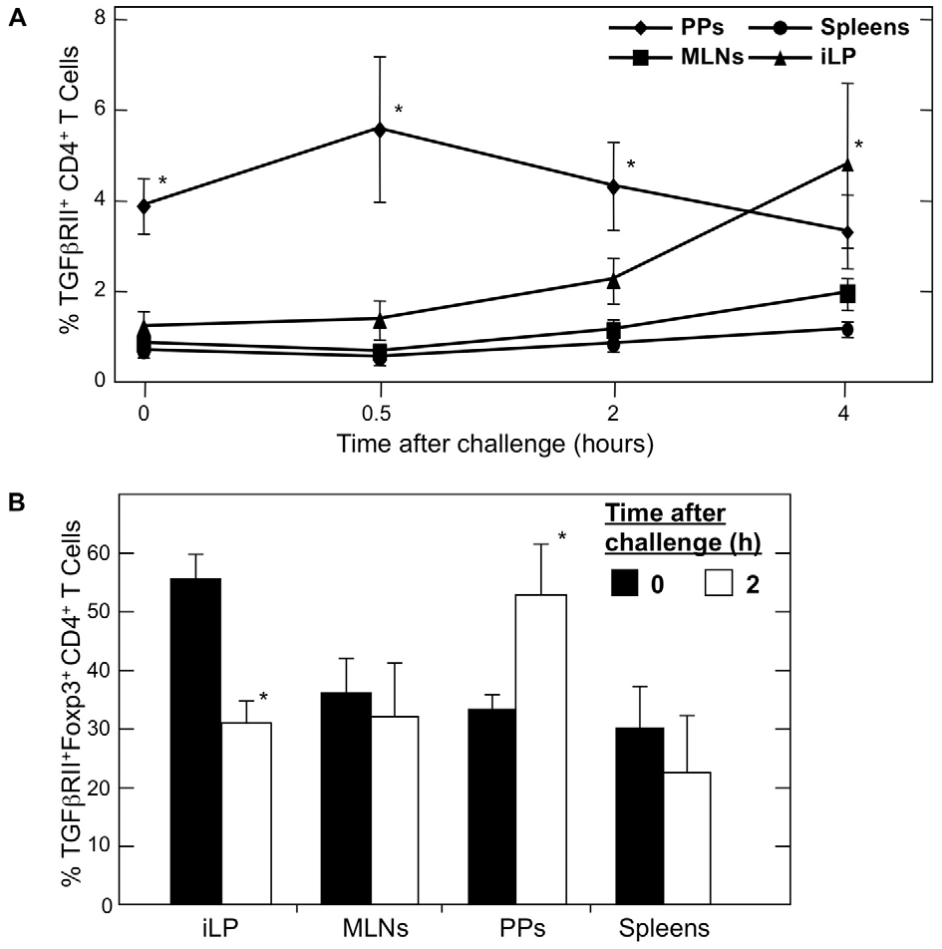
Frequency of CD4^+^ TGFβRII^+^ T cells in tissues of tolerized C57BL/6 mice after single challenge with OVA plus CT. *A*. Mononuclear cells were isolated from PPs, MLNs, iLP and spleens of C57BL/6 mice 7 days after 30 mg of OVA feeding (0) and at 0.5, 2 and 4 h after single challenge with 1 mg of OVA plus 10 µg of CT (OVA plus CT). Cells were then stained with FITC-conjugated anti-hTGFβRII, APC-labeled anti-CD4, and biotin-tagged anti-CD3 mAbs followed by PerCP-Cy™5.5-conjugated streptavidin. *B.* Cells were isolated as described above and stained with FITC-conjugated anti-hTGFβRII, APC-labeled anti-CD4, PE-labeled anti-hFoxp3, and biotin-tagged anti-CD3 mAbs followed by PerCP-Cy™5.5-conjugated streptavidin. Samples were subjected to flow cytometry analysis by FACSCalibur®. *n* = 5 mice per time point, *p<0.05 compared to sham-tolerized and OVA plus CT challenged controls.

Following a typical oral challenge protocol of three weekly feedings of 1 mg of OVA plus 10 µg of CT (OVA plus CT) starting one week after large-dose tolerance induction, we found that there was no significant difference in expression of TGFβRII on CD4^+^ T cells between mucosal and systemic lymphoid tissues. The frequency of CD4^+^ TGFβRII^+^ T cells in the PPs, MLNs, iLP and spleen had returned to basal levels by one week after the final challenge (data not shown). Upon further analysis, this drop in expression was observed by 24 h after a single oral challenge with OVA plus CT. Therefore, we focused on early time points after the first oral challenge in order to examine the kinetics of this observed decrease. Within 30 min of OVA plus CT administration, numbers of CD4^+^ TGFβRII^+^ T cells decreased in the PPs (Fig. 3A). Mice fed PBS showed essentially no change in CD4^+^ TGFβRII^+^ T cells after oral challenge. When TGFβRII expression in the iLP was also examined in mice fed OVA, a significantly increased number of CD4^+^ TGFβRII^+^ T cells were seen 4 h following oral challenge. Further, MLNs and spleen showed increased numbers of CD4^+^ TGFβRII^+^ T cells. These data suggest that the CD4^+^ TGFβRII^+^ T cells may be dispatching from the PPs to effector tissues via the draining lymph nodes shortly after challenge. Importantly, by two hours after oral challenge with OVA plus CT, CD4^+^ TGFβRII^+^ T cells from the PPs of mice fed a large dose of OVA have significantly increased intracellular Foxp3 (Fig. 3B). In contrast, CD4^+^ TGFβRII^+^ Foxp3^+^ T cells decreased in iLP, MLNs and spleen during this time. Further, Foxp3 expression in CD4^+^ TGFβRII^-^ T cells decreased in all tissues over this period of time (data not shown). It is interesting to note that these results also indicate a direct affect of OVA plus CT challenge on CD4^+^ T cells, since this time frame would preclude Ag processing and presentation by APCs.

### PPs have increased frequencies of regulatory cytokine-producing CD4^+^ T cells after induction of oral tolerance and single oral challenge with OVA plus CT

**Figure 4:**
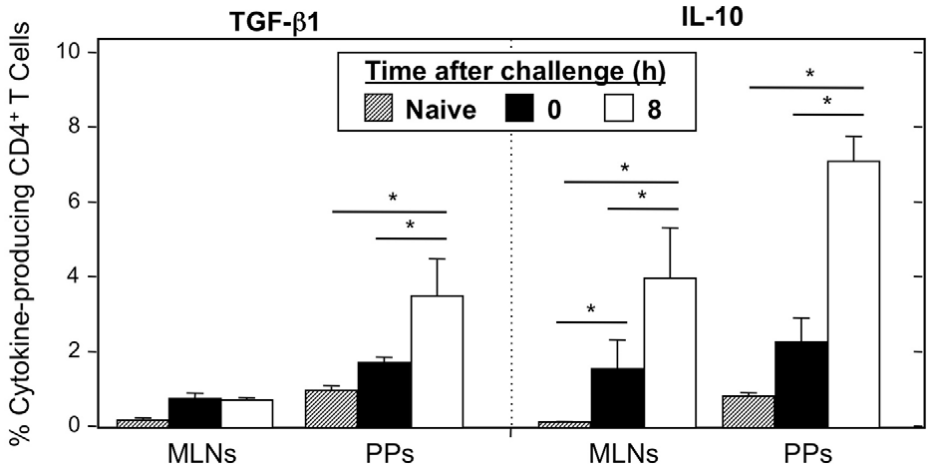
Regulatory cytokine production by CD4^+^ T cells of naïve and tolerized C57BL/6 mice before and after single challenge with OVA plus CT. Mononuclear cells were isolated from PPs and MLNs of naive C57BL/6 mice and from mice 7 days after 30 mg of OVA feeding (0) and 8 h after single oral challenge with 1 mg of OVA plus 10 µg of CT (OVA plus CT). Cells were then stained with FITC-conjugated anti-hTGFβRII, APC-labeled anti-CD4, and biotin-tagged anti-CD3 mAbs followed by PerCP-Cy™5.5-conjugated streptavidin. Cells were stained with APC-labeled anti-CD4, PE-labeled anti-IL-10 or anti-hTGF-β1, and biotin-tagged anti-CD3 mAbs followed by PerCP-Cy™5.5-conjugated streptavidin. Samples were subjected to flow cytometry analysis by FACSCalibur®. *n* = 4 mice per time point, *p<0.05.

CD4^+^ T cells from MLNs and PPs of tolerized C57BL/6 mice before and after single challenge with OVA plus CT, as well as naïve mice, were analyzed for the presence of the suppressive cytokines IL-10 and TGF-β1. Cells from naïve mice showed very low expression of these cytokines (Fig. 4). Seven days after the mice were fed a large dose of OVA, the frequency of CD4^+^ TGF-β1^+^ or IL-10^+^ cells had increased in both the MLNs and PPs. However, in the MLNs, the increase in TGF-β1 was negligible. Within 8 hours after administering OVA plus CT to the tolerized mice, the frequency of TGF-β1^+^ and IL-10^+^ T helper cells increased significantly in the PPs. While MLNs have a similar increase in the frequency of CD4^+^ IL-10^+^ T cells after tolerance plus single oral challenge, the magnitude of this increase is less than that seen in PPs. Further, unlike PPs, MLN CD4^+^ T cells did not show an increase in TGF-β1. These two points indicate that the PPs are sites of preferential up-regulation of T cells with regulatory capabilities after oral tolerance induction.

### Abrogation of TGFβRII signaling in CD4^+^ T cells fails to elicit mucosal unresponsiveness

**Figure 5:**
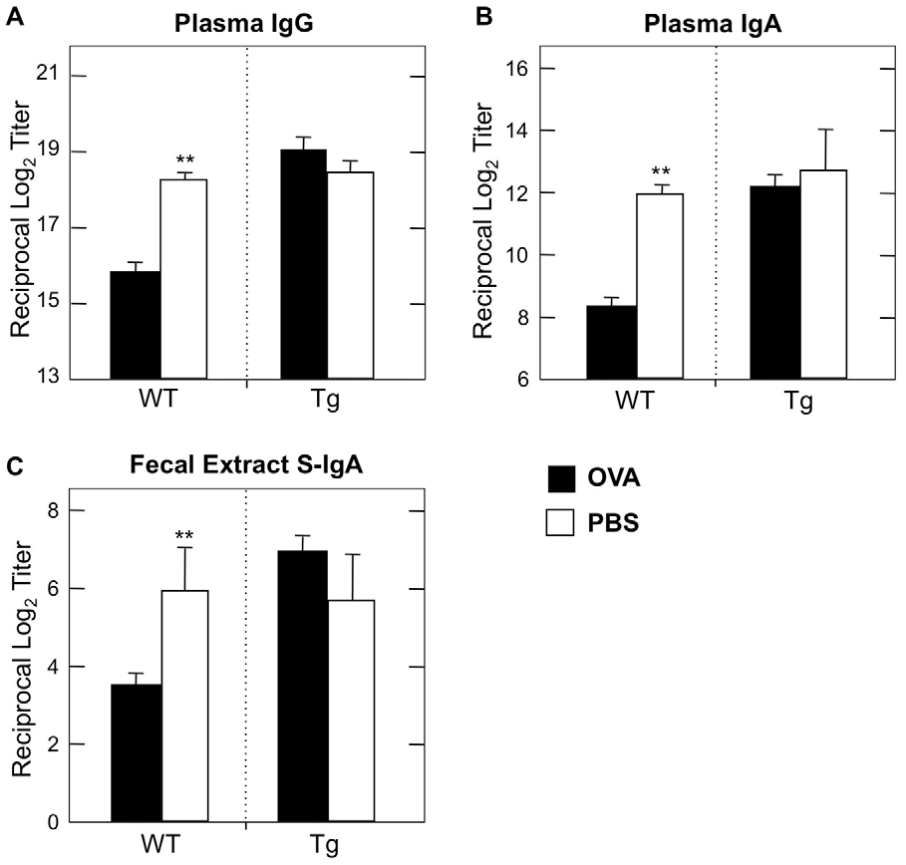
OVA-specific Ab responses in CD4dnTGFβRII mice after large-dose oral tolerance and oral challenge with OVA plus CT. *A-C.* One week after the last challenge, plasma and fecal extracts were subjected to OVA-specific ELISA to determine IgG, IgA (plasma; *A, B*) or S-IgA (fecal extract; *C*). Endpoint titers are expressed as the last dilution yielding OD_415_ of > 0.1 units above background control values. For CD4dnTGFβRII experiments: *n* = 8 OVA-fed, *n* = 5 PBS controls. For wild-type experiments: *n* = 19 OVA-fed, *n* = 8 PBS controls. **p<0.001.

**Figure 6:**
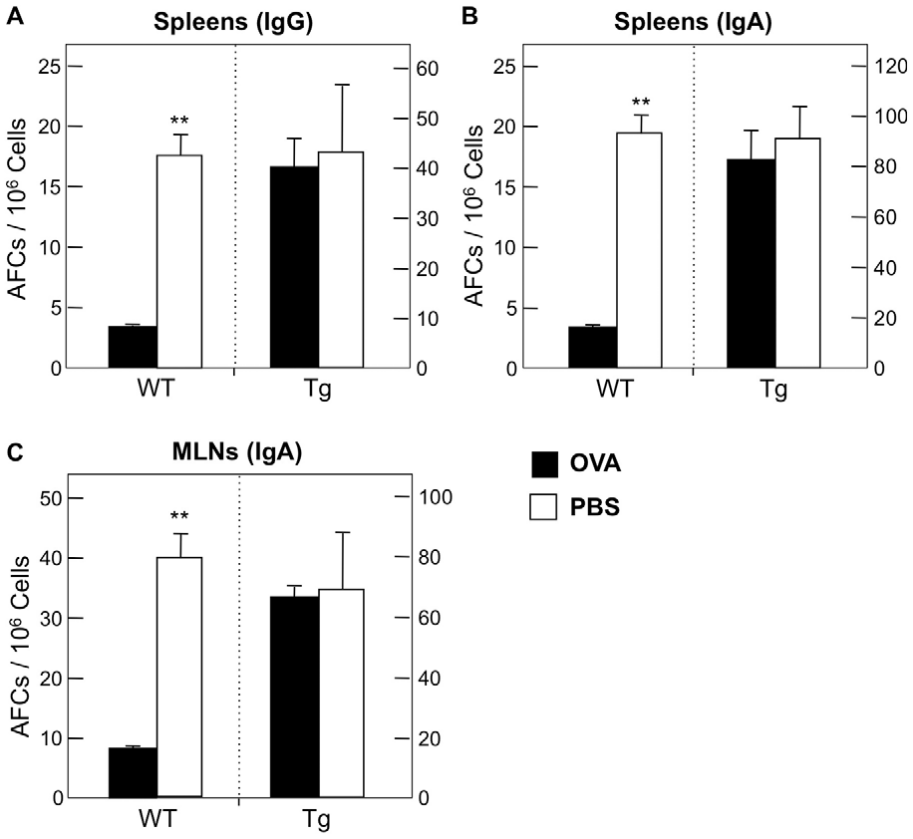
OVA-specific Ab Forming Cells (AFCs) in CD4dnTGFβRII mice after large-dose oral tolerance and oral challenge with OVA plus CT. *A-C.* Mononuclear cells were isolated from MLNs and spleens one week after the final challenge. Cells were then subjected to an ELISPOT assay to detect numbers of OVA-specific AFCs. For CD4dnTGFβRII experiments: *n* = 8 OVA-fed, *n* = 5 PBS controls. For wild-type experiments: *n* = 19 OVA-fed, *n* = 8 PBS controls. **p<0.001.

Our findings thus far indicate that TGFβRII expression by CD4^+^ T cells is influenced by large- and small-dose Ag feeding. However, it is important to determine whether the function of this receptor is required for oral tolerance induction. To this end, we next examined the role of functional TGFβRII signaling in the induction of oral tolerance in a mouse model. For these experiments, we utilized CD4dnTGFβRII mice in which TGF-β1 signaling is abrogated in all CD4^+^ T cells. Wild-type littermates served as controls. CD4dnTGFβRII mice fed 30 mg of OVA prior to oral challenge show no significant decrease in OVA-specific plasma IgG and IgA Ab responses when compared with those of mice fed PBS (Fig. 5A, B). Thus, the levels of anti-OVA IgG and IgA Abs in plasma were essentially the same as those Ab responses seen in wild-type mice fed PBS (Fig. 5A, B). In contrast, plasma of wild-type mice fed OVA contained significantly reduced levels of OVA-specific IgG and IgA Ab responses. These results were further confirmed at the cellular level. Thus, numbers of anti-OVA IgG and IgA AFCs in spleen of CD4dnTGFβRII mice were not significantly different from PBS-fed CD4dnTGFβRII mice (Fig. 6A, B).

Since this oral OVA plus CT challenge system is designed for examining unresponsiveness in mucosal effector tissues, we next determined OVA-specific S-IgA Ab responses in the GI tract of mice fed OVA. CD4dnTGFβRII mice fed 30 mg of OVA prior to oral challenge with OVA plus CT showed essentially no reduction in OVA-specific S-IgA Ab responses in fecal extracts (Fig. 5C). Further, numbers of anti-OVA IgA AFCs in MLNs of CD4dnTGFβRII mice fed OVA were comparable to those of PBS-fed CD4dnTGFβRII mice (Fig. 6C). On the other hand, wild-type mice fed OVA showed significant reductions in OVA-specific S-IgA Ab responses in fecal extracts (Fig. 5C) and MLNs when compared with those of PBS-fed wild-type mice (Fig. 6C). These data clearly indicate that no down-regulation of OVA-specific mucosal and systemic Ab responses were induced in mice without functional TGFβRII expression by CD4^+^ T cells.

### DTH responses are not reduced in OVA-fed CD4dnTGFβRII mice

**Figure 7:**
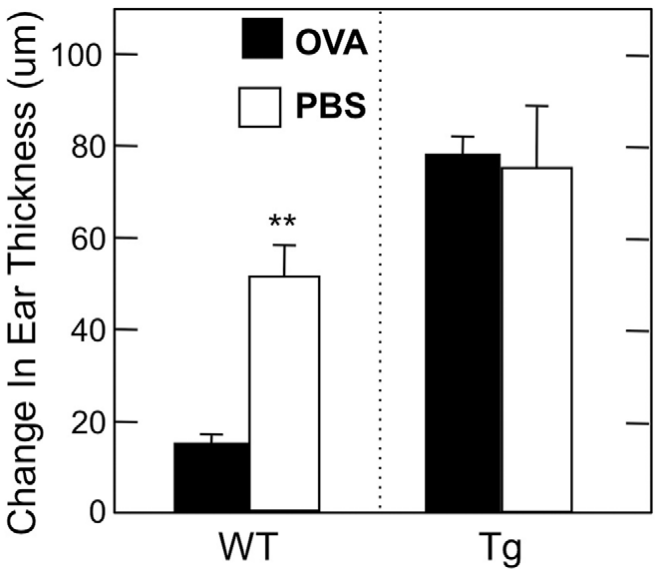
DTH responses in CD4dnTGFβRII mice after large-dose oral tolerance and oral challenge with OVA plus CT. Six days after final immunization, the ear pinnae of mice were injected with 20 µg OVA or PBS. Ear swelling was measured 24 h later. The DTH response is expressed the difference in thickness between left and right ears. For CD4dnTGFβRII experiments: *n* = 6 OVA-fed, *n* = 6 PBS controls. For wild-type experiments: *n* = 19 OVA-fed, *n* = 4 PBS controls. **p<0.001.

In order to further confirm lack of oral tolerance induction in CD4dnTGFβRII mice at the T cell level, we assessed OVA-specific DTH responses in PBS- and OVA-fed CD4dnTGFβRII and wild-type mice. CD4dnTGFβRII mice fed 30 mg OVA showed robust DTH responses 24 h after the challenge. This response was similar to DTH responses seen in both PBS-fed CD4dnTGFβRII and wild-type mice and was significantly greater than the response of 30 mg OVA-fed C57BL/6 mice (Fig. 7). Combined with our Ag-specific antibody data, these results further underline the importance of intact TGF-β1 signaling in CD4^+^ T cells for mucosal and systemic unresponsiveness.

## Discussion

In this study we showed that within 24 h of inducing oral tolerance, TGFβRII expression by CD4^+^ T cells in the PPs is significantly upregulated. This upregulation was independent of fed Ag dosage. This receptor is part of the oligomeric receptor complex that recognizes TGF-β1 and it has been shown that this molecule is required for TGF-β1 signaling [30]. Many studies have shown that TGF-β1 plays a central role in the development of acquired-type Ag-specific Tregs, through the induction and maintenance of Foxp3 expression in CD25^-^ and CD25^+^ T cells, respectively [25]-[27], [46]. Therefore, the up-regulation of TGFβRII by CD4^+^ T cells indicates an increased ability to respond to TGF-β1 and could therefore predispose these cells to differentiate into Ag-specific Tregs [24].

After administering a high-dose oral tolerance induction protocol (i.g. 30 mg OVA), we found that CD4^+^ T cells in the PPs maintained a high frequency of TGFβRII expression. CD4^+^ T cells from other lymphoid tissues either did not increase TGFβRII expression, or did not maintain an elevated expression of the receptor–as in the spleen or MLNs and iLP. TGFβRII analysis during a low-dose oral tolerance induction protocol (*ad lib* 1 mg/ml OVA in water) confirmed that TGFβRII is upregulated by PP CD4^+^ T cells regardless of the magnitude of the dose of OVA. Further, MLN and splenic CD4^+^ T cells failed to upregulate TGFβRII expression and iLP did not maintain upregulation during the low-dose protocol. These novel findings indicate that PPs are uniquely populated with CD4^+^ T cells that are primed to respond to TGF-β1 after a tolerogenic event.

When tolerized mice were orally challenged after seven days with OVA plus CT, expression of TGFβRII on CD4^+^ T cells rapidly decreased in the PPs and increased in the MLNs and iLP. These data indicate that after challenge, this cell population may mobilize and migrate into other mucosal inductive and effector sites. Interestingly, by 2 h after a single OVA plus CT challenge, Foxp3 expression in PP CD4^+^ TGFβRII^+^ T cells nearly doubles, but decreases in the other tissues by up to 50%. In contrast, Foxp3 decreases in CD4^+^ TGFβRII^-^ cells over this period of time (data not shown). This finding shows that the increased expression of TGFβRII leads to preferential upregulation of Foxp3 in this cell population. Interestingly, the significant increase in frequency of IL-10- and TGF-β1-producing CD4^+^ T cells in PPs after oral tolerance induction and single oral challenge further show that the PPs are sites of significant regulatory capacity, including the production of suppressive cytokines that are known to induce differentiation of Tregs [25], [47]. Taken together these findings indicate that the PPs are playing an important, non-redundant role in oral tolerance induction by being suitable sites for TGF-β1-dependent, Ag-specific Treg differentiation.

We next examined the role of this receptor in oral tolerance by using a large dose of OVA in CD4dnTGFβRII mice. These mice express a signaling deficient TGFβRII molecule on all T cells and therefore these cells fail to respond to TGF-β1 [35], [36]. The importance of TGF-β1 in the peripheral differentiation of naïve T cells into Treg cells has been described (34-36). Indeed, a recent study found that reduced expression of TGFβRII was correlated with impaired differentiation of peripheral (Ag-specific) Tregs (37). When compared to wild-type mice, CD4dnTGFβRII mice have an increased frequency of CD4^+^ TGFβRII^+^ T cells. This increase is due to the loss of function of the receptor. However, despite this increased expression, when young adult CD4dnTGFβRII mice were fed a 30 mg dose of OVA prior to oral challenge with OVA plus CT, oral tolerance could not be induced. Thus, these mice revealed high levels of Ag-specific IgG and IgA Ab responses in plasma and fecal extracts which were comparable to PBS fed CD4dnTGFβRII mice challenged with OVA plus CT. Further, the OVA-specific DTH response was not reduced in CD4dnTGFβRII mice fed OVA. These novel findings indicate that both expression and proper function of TGFβRII are critical to oral tolerance induction. The lack of oral tolerance induction in CD4dnTGFβRII mice is most likely not due to the loss of Treg cell development but to the absence of TGF-β1 signaling in effector CD4^+^ T cells. To support this view, it has been shown that TGF-β1 producing cells were not increased in mice orally tolerized with OVA [44], [48], [49]. In order to further confirm this point, we are currently testing whether adoptive transfer of OVA-induced Treg cells induce systemic unresponsiveness in CD4dnTGFβRII mice.

The role of PPs in the induction of oral tolerance has proved to be a controversial subject. Our previous studies showed that PPs are indeed required for the development oral tolerance to protein Ags. Thus, mice treated *in utero* with lymphotoxin (LT) βR-Ig, and were therefore PP-null, could not develop oral tolerance to OVA [37]. In response to this study, another group reported that PP-null mice could be induced to oral tolerance and, further, that MLNs were instead the key organ for oral tolerance induction [39], [40]. Although the MLNs are the immunological portal for nearly all gut-derived Ags [1], it is more likely that the PPs and MLNs work together during a tolerance event, since recent evidences for the importance of PPs in oral tolerance have come to light. Thus, it was shown that Ag-specific Tregs can be cloned from the PPs of orally-tolerized mice [23], while others found that PPs are required for oral tolerance to experimental autoimmune encephalitis in mice following myelin basic protein feeding [50]. Further, our recent work indicates that by targeting the M-cells of the follicle-associated epithelium of PPs using Ag fused with the s1 protein of reovirus, the dosage of Ag needed to induce oral tolerance is reduced by 1000 fold [38]. The present study shows that there is not a significant difference in the frequency of TGF-β1- or IL-10-producing CD4 T cells in the PPs and MLNs seven days after tolerance is induced. However, we show that once the mice are challenged with the antigen, the PPs are sites of significant upregulation of these cytokines, more so than MLNs. These findings are seemingly in contrast with previous studies showing that TGF-β1 does not play a role in large-dose oral tolerance [44], [48], [49], however these studies examined TGF-β1 production at the end of oral tolerance induction and systemic or mucosal challenge protocol. The present study focuses on early time points after a single oral challenge with antigen and adjuvant and therefore shows that TGF-β1 plays a role in the initiation of large-dose oral tolerance rather than in the execution of this response at later time points. Therefore our findings further underscore the importance of PPs in the very early time points of oral tolerance induction by showing that these tissues are populated with T helper cells that are primed to respond to TGF-β1 of this response and that these cells mobilize shortly after re-exposure to the tolerizing Ag.

Indeed, our study further adds to the growing evidence that the two traditional modes of oral tolerance are linked. Early studies on the mechanisms of oral tolerance have led to the development of a dogma that how oral tolerance is induced, mediated and maintained is determined by the size and frequency of Ag dose [1]-[3], [19]. It is commonly held that large doses of protein Ag cause anergy and/or deletion of Ag-specific T cells, while small frequent doses or continual exposure to Ag induce the differentiation of regulatory T cells that mediate suppression by the Ag-specific production of suppressive cytokines [19]. However, more recent studies have found that these are not mutually exclusive occurrences; that Ag-specific Tregs can be found after feeding large doses of Ag [13], [23] and that anergy may also play a role in oral tolerance to small doses of Ag [13]. The increase in TGFβRII expression on CD4^+^ T cells in PPs could indicate that these cells are primed to differentiate into Treg cells [24], [51]. Finally, our results may indicate an increase in the necessity for CD4^+^ T cells to respond to TGF-β1 after large doses of Ag, indicating a link between active suppression and this form of oral tolerance.

In conclusion, our current study shows that significant and persistent increases in TGFβRII expression on CD4^+^ T cells occur in the PPs immediately after tolerizing events, independent of Ag dose. When challenged these PP CD4^+^ TGFβRII^+^ cells upregulate Foxp3 expression. Expression of the TGFβRII alone is insufficient, as is indicated by the lack of oral tolerance induction in mice lacking functional TGFβRII on CD4^+^ T cells even in the presence of increased expression of the receptor. These increases may indicate that the PPs are important sources of tolerogenic Tregs that begin differentiating in early time points after Ag feeding. Further study is needed to determine if these cells are indeed Tregs or perhaps precursors of Tregs, and to examine the mechanism of their development and function after challenge with Ag and mucosal adjuvant. To this end, experiments to determine the function of these PP-derived CD4^+^ TGFβRII^+^ T cells are currently being undertaken in our laboratory.

## Supporting Information

Figure S1 Positive controls for FITC-hTGFβRII mAb. Mononuclear cells were isolated from MLNs and PPs of naïve CD4dnTGFβRII mice. Cells were then stained with FITC-conjugated anti-hTGFβRII, APC-labeled anti-CD4, and biotin-tagged anti-CD3 mAbs followed by PerCP-Cy™5.5-conjugated streptavidin. Analysis is gated on CD4^+^ T cells. Representative FACS plots are shown.
(TIF)
