# Supplementary figures and images for: Functional Transforming Growth Factor-β Receptor Type II Expression by CD4+ T Cells in Peyer's Patches Is Essential for Oral Tolerance Induction
Source: PLoS One. 2011 Nov 7;6(11):e27501. doi: 10.1371/journal.pone.0027501 (PMC3210179; doi:10.1371/journal.pone.0027501)

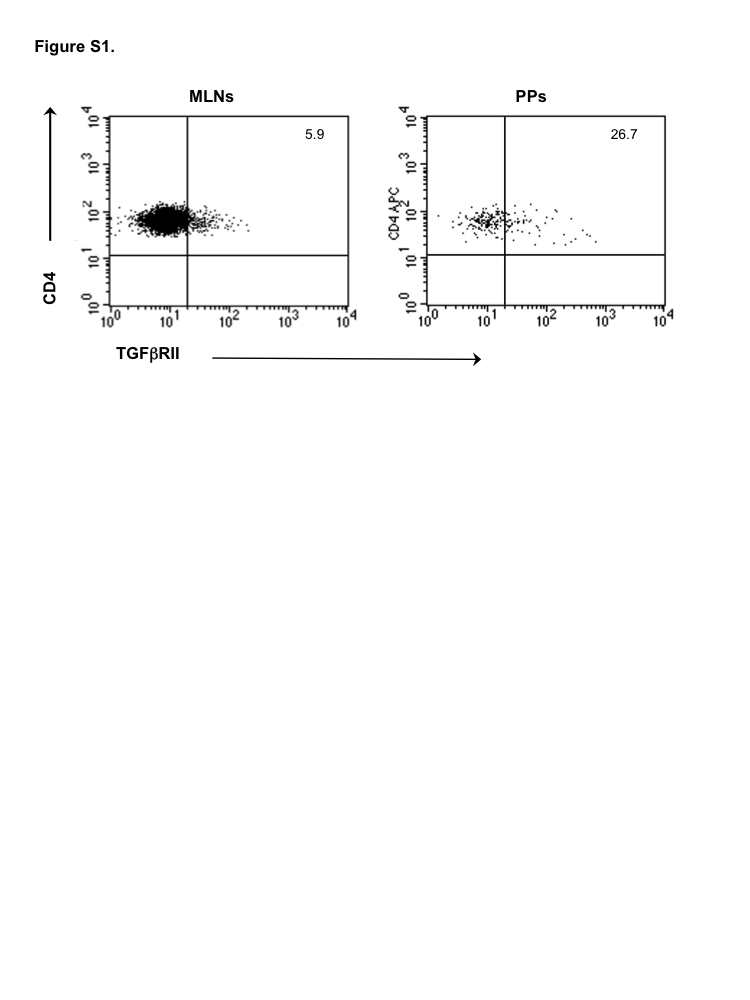

Supplement: Figure S1 — Positive controls for FITC-hTGFβRII mAb. Mononuclear cells were isolated from MLNs and PPs of naïve CD4dnTGFβRII mice. Cells were then stained with FITC-conjugated anti-hTGFβRII, APC-labeled anti-CD4, and biotin-tagged anti-CD3 mAbs followed by PerCP-Cy™5.5-conjugated streptavidin. Analysis is gated on CD4+ T cells. Representative FACS plots are shown. (TIF) [file pone.0027501.s001.tif]
